# Influenza Epidemiology in Finland During and After the COVID‐19 Pandemic: Surveillance Data Analysis (2019–2024)

**DOI:** 10.1111/irv.70131

**Published:** 2025-06-17

**Authors:** Ulrike Baum, Niina Ikonen, Oskari Luomala, Eero Poukka, Tuija Leino, Hanna Nohynek

**Affiliations:** ^1^ Department of Public Health Finnish Institute for Health and Welfare Helsinki Finland; ^2^ Department of Public Health, Faculty of Medicine University of Helsinki Helsinki Finland

**Keywords:** COVID‐19 pandemic, Finland, influenza, influenza vaccines

## Abstract

**Background:**

The Finnish influenza surveillance system combines traditional virological surveillance and analyses of electronic health records. This paper describes the influenza epidemiology in Finland (population: 5.5 million) during and after the COVID‐19 pandemic based on national surveillance data from 2019 to 2024.

**Methods:**

Influenza incidence was evaluated based on three register‐based outcomes: laboratory‐confirmed infections, primary health care visits, and hospitalizations. Virus‐type distributions were analyzed from respiratory specimens. In register‐based analyses, vaccination coverage and vaccine effectiveness were assessed for the two cohorts universally included in the Finnish vaccination program: children aged ≤ 6 years and adults aged ≥ 65 years.

**Results:**

The 2019/2020 influenza epidemic ended with the introduction of COVID‐19 containment measures. In 2020/2021, influenza was largely absent. The 2021/2022 epidemic peaked exceptionally late. Influenza activity returned to prepandemic levels in 2022/2023. None of the 717 sentinel specimens tested positive for B/Yamagata. Although the percentage of vaccinated young children was constant (31% [100,387/323,614] to 37% [126,984/346,344]), the percentage of vaccinated elderly people increased from 48% (577,404/1,211,732) in 2019/2020 to 63% (787,771/1,255,644) in 2021/2022. The vaccine effectiveness against hospitalization due to laboratory‐confirmed influenza in young children and elderly people was 68% (95% confidence interval: 38%; 83%) and 42% (34%; 50%) in 2022/2023, respectively, and slightly lower in 2023/2024.

**Conclusions:**

The COVID‐19 pandemic had two potentially lasting effects on influenza: elimination of the B/Yamagata lineage and improved vaccination coverage in the elderly population in Finland. To strengthen the Finnish influenza surveillance system, participation in sentinel surveillance must be improved.

## 1 Introduction

Influenza is a worldwide circulating respiratory disease caused by a quickly evolving virus. To combat influenza, broadscale vaccination programs exist. More than 100 countries have established surveillance systems or national influenza centers to track the virus's evolution and to evaluate and guide public health interventions [1].

Finland, a Nordic country with a population of about 5.5 million, has a hybrid influenza surveillance system combining traditional virological surveillance and analyses of electronic health records [2]. The Finnish influenza surveillance system enables the identification of the start of an influenza epidemic and the real‐time monitoring of its progress, the genetic characterization of the circulating influenza viruses, and the surveillance of drug resistances [2]. In addition, the system facilitates the evaluation of disease burden, morbidity and mortality, the real‐time monitoring of influenza vaccine uptake, and the timely estimation of influenza vaccine effectiveness [2].

From 2013 to 2019, an annual influenza surveillance report was published by the Finnish Institute for Health and Welfare (THL) at the end of each season [3]. Due to the disruptive effects of the coronavirus disease 2019 (COVID‐19) pandemic, no such surveillance report has been published through March 2025. Without the intention to replace the resumed annual reporting, it is therefore the objective of this paper to describe trends in the epidemiology of influenza during and shortly after the COVID‐19 pandemic in Finland based on surveillance data from 2019 to 2024. In particular, this paper presents the Finnish influenza surveillance system, outlines the course and effects of the COVID‐19 pandemic, examines the influenza incidence and distribution of virus types, and summarizes the estimates of vaccination coverage and vaccine effectiveness.

## 2 Methods

### 2.1 The Finnish Influenza Surveillance System

The Finnish influenza surveillance system is based on laboratory analyses of respiratory specimens, on communicable disease notifications filed by laboratories, recorded in the National Infectious Diseases Register, as well as on data about primary health care visits, hospitalizations, and vaccinations recorded in the Register of Primary Health Care Visits, the Care Register of Health Care, and the National Vaccination Register, respectively. All register data are available in real time and can be linked using a unique person identifier. The access to that person identifier is, however, strongly restricted; pseudonymized identifiers are used instead to mitigate data security risks. The data are routinely analyzed by THL under the provisions of the Communicable Diseases Act, which allows the use of electronic health records for monitoring and controlling infectious diseases such as influenza without individual consent. The results are published as aggregated open data, communicated through press releases, and confidentially shared with national and international partners, such as the Ministry of Social Affairs and Health, the National Advisory Committee on Vaccines, and the World Health Organization. This paper presents a summary of these results.

### 2.2 Clinical Influenza Surveillance

In Finland, seasonal influenza is mainly diagnosed in the primary health care sector based on clinical criteria. In general, the laboratory confirmation of mild cases is considered not necessary if more than 48 h have passed since symptom onset [4]. Severe cases, such as those requiring hospitalization, and severely immunocompromised cases are, however, recommended to be laboratory confirmed and, if necessary, given antiviral medication.

To evaluate the overall influenza incidence, three metrics, which collectively provide a comprehensive overview of the influenza disease burden on the Finnish health care system, were used: the incidence of laboratory‐confirmed influenza infection, the incidence of primary health care visits due to influenza‐like illness (ILI), and the incidence of hospitalization due to influenza infection.

The number of laboratory‐confirmed infections was taken from the National Infectious Diseases Register, which records the sample dates and virus types of all influenza‐positive specimens analyzed in Finnish clinical microbiology laboratories. Selecting only visits due to ILI (ICD‐10: J09–J11 or ICPC‐2: R80), the number of primary health care visits was taken from the Register of Primary Health Care Visits. That register records the dates and diagnoses of all public (since 2012) and all occupational (since 2020) primary health care visits, as well as of many private primary health care visits (since 2020). Linking Care Register for Health Care and National Infectious Diseases Register data, hospitalization due to influenza infection was defined as hospitalization with respiratory disease (ICD‐10: J00–J22, J46, J80–J84, J85.1, J86, and U07) up to 7 days before or 14 days after a laboratory‐confirmed influenza infection. The Care Register for Health Care records the dates and diagnoses of visits and stays in hospitals, day surgeries, and emergency rooms.

In addition to the three overall metrics, age‐specific incidence rates were calculated by dividing the weekly number of cases by the weekly population size from the Population Information System. The study period was from August 2019 to May 2024 split into influenza seasons from August to July. However, as this analysis is solely based on THL's open data [5, 6, 7], hospitalization counts were only available from January 2020.

### 2.3 Virological Influenza Surveillance

Traditional virological surveillance in Finland revolves around laboratory analyses of respiratory samples at THL. Since 2012, outpatient sentinel sites have been recruited to collect three to five respiratory specimens (nasopharyngeal swabs) per week from volunteering patients with ILI or an acute respiratory infection. In addition, intensive care units have been requested to send 5–10 respiratory specimens (nasopharyngeal swabs, suction mucus samples, or lavage samples from the nasopharynx, trachea, or lungs) from volunteering patients with severe acute respiratory infection. The syndromic case definitions used in Finland for virological influenza surveillance strictly adhere to the clinical criteria set by the Council of the European Union, such as sudden onset of symptoms and the presence of at least one respiratory symptom [8].

Taking into account geographic distribution, population structure, and staff commitment, the site selection has aimed for high representativeness, while relying on voluntariness. In 2019–2020, there were 16 participating sentinel sites, representing public and private general practitioners as well as garrisons, and 13 participating intensive care units. Due to difficulties in recruiting sites since the start of the COVID‐19 pandemic, the virological surveillance of influenza viruses was interrupted in 2020–2021 and continued thereafter with a decreased and varying number of sites: Only seven sentinel sites participated in 2021–2022, and a maximum of two intensive care units have participated since 2022–2023. To adjust for that also, clinical microbiology laboratories have been requested to send influenza‐positive specimens, although their samples are unlikely representative.

The laboratory analysis of all specimens, collected or requested for virological surveillance, takes place at THL. The samples are tested for influenza A and B viruses, among other respiratory pathogens, using real‐time polymerase chain reaction. This method of detecting viral nucleic acid ensures the sensitive and specific identification of the influenza virus type, subtype, and lineage. Furthermore, the genes encoding the hemagglutinin and neuraminidase surface proteins as well as the polymerase acidic protein are sequenced in some specimens to investigate genetic and antigenic changes; this aspect, however, is beyond the scope of this paper, and the reader is referred to the resumed annual reporting [3].

To evaluate the distribution of virus types in each season, the number and proportion of influenza‐positive specimens collected between Calendar Weeks 40 and 20 (26 in 2021–2022) by sentinel sites and intensive care units or sent by clinical microbiology laboratories were recorded by subtype and lineage. In 2020–2021, no samples were taken. Specimens not typable due to low virus concentration were excluded. Specimens positive for more than one subtype or lineage were counted as multiple specimens.

### 2.4 Influenza Vaccine Uptake and Vaccination Coverage

To protect against influenza and its sequelae, seasonal influenza vaccination is recommended to more than half of the Finnish population. As part of the national vaccination program, funded by the Finnish government and implemented by Finland's 21 wellbeing services counties, seasonal influenza vaccination is offered free of charge to children aged 6 months to 6 years (hereinafter referred to as children younger than 7 years), adults aged 65 years and above, military conscripts, pregnant women, social and health care workers, people in institutional settings, people at risk of severe influenza disease because of an underlying chronic illness or immunosuppressive treatment, and people close to a person at particularly high risk of severe influenza disease [9]. Since 2023, the wellbeing services' counties may also give seasonal influenza vaccines from the national vaccination program to people working on fur farms, with poultry, official veterinarians, laboratory workers handling avian influenza samples, and others at high risk of avian influenza [9]. Additionally, occupational and public and other private primary health care services provide seasonal influenza vaccination outside the national vaccination program at the cost of the employer or individual.

Everyone covered by the program is offered an inactivated influenza vaccine injected into a muscle. However, children aged 2–6 years may choose a live‐attenuated vaccine administered as nasal spray instead.

To monitor the influenza vaccine uptake, that is, the progress of the seasonal vaccination campaigns, the weekly number of influenza vaccinations was taken from the National Vaccination Register, which records the dates and brands of all vaccinations given during visits or stays documented in the Register of Primary Health Care Visits and, since 2019, the Care Register for Health Care. In addition, age‐specific vaccination coverage figures were calculated for children younger than 7 years and adults aged 65 years and above, the two age groups universally included in the national vaccination program, by dividing the number of vaccinated individuals by the population size from the Population Information System. The study period was from August 2019 to May 2024, split into influenza seasons from August to July.

### 2.5 Influenza Vaccine Effectiveness

Influenza vaccine effectiveness describes the average direct protective effect that an individual gains by influenza vaccination. In a partially vaccinated population like the Finnish one, it can be estimated by comparing the influenza incidence in vaccinated and unvaccinated people.

In each of the past five seasons except for 2020–2021, age‐specific influenza vaccine effectiveness was routinely assessed for children younger than 7 years and adults aged 65 years and above. In register‐based time‐to‐event analyses, each age group formed a nationwide cohort whose individuals were followed through an influenza season, as previously detailed [10]. The study period for each influenza season ran between Calendar Weeks 40 and 20, except for 2021–2022, which extended to June 30, 2022. Exposure and outcome of interest were the first influenza vaccination and the first laboratory‐confirmed infection with any influenza virus in the season. In sensitivity analyses, the exposure was stratified by time since vaccination and vaccine type. In 2022–2023 and 2023–2024, a secondary outcome was included: hospitalization with respiratory disease (ICD‐10: J00–J22, J46, J80–J84, J85.1, and J86) up to 2 days before or 7 days after a laboratory‐confirmed infection with any influenza virus.

Excluding the first 14 postvaccination days, the vaccine effectiveness was estimated as 1 minus the hazard ratio adjusted either for calendar year of birth (when studying young children) or for age, sex, influenza vaccination history, nights hospitalized in the previous 5 years, and presence of underlying chronic conditions (when studying the elderly population). The suitability of the latter set of covariates to control for confounding had been demonstrated in a previous study [11]. The hazard ratio was assumed to be constant over a season and estimated using Cox regression with calendar time as the underlying time scale.

### 2.6 The Course of the COVID‐19 Pandemic in Finland

To outline country‐specific events that occurred during the study period, the results start with a summary of the course of the COVID‐19 pandemic in Finland and the Finnish pandemic response. In analogy to clinical influenza surveillance and influenza vaccine uptake, the corresponding COVID‐19 figures are presented. The clinical case definitions were laboratory‐confirmed infection with severe acute respiratory syndrome coronavirus 2 (SARS‐CoV‐2), the etiological agent of COVID‐19, and hospitalization with respiratory disease (ICD‐10: J00–J22, J46, J80–J84, J85.1, J86, and U07) up to 7 days before or 14 days after a laboratory‐confirmed infection.

## 3 Results

### 3.1 The Course of the COVID‐19 Pandemic in Finland

**FIGURE 1:**
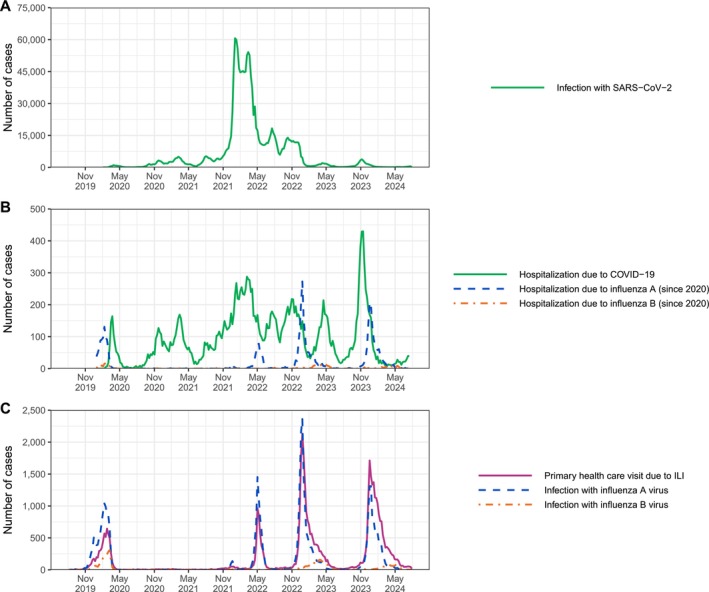
COVID‐19 and influenza incidence in Finland by calendar week, August 2019 to May 2024. (A) Infection with SARS‐CoV‐2; (B) Hospitalization due to COVID‐19 and influenza; (C) Primary health care visit due to ILI, and infection with influenza virus. Adapted from [4, 5, 6].

In Finland, the first documented COVID‐19 case was detected in late January 2020 [12]. The following first wave of COVID‐19 rose and receded during the first half of 2020 and was flat in comparison to the later waves 2020–2021. With the emergence of the Omicron variant in 2022 [13], case numbers exploded, but the disease severity substantially decreased. In July 2022, COVID‐19 was proclaimed endemic in Finland [14] with seasonal epidemics occurring afterwards (Figure 1A,B).

**FIGURE 2:**
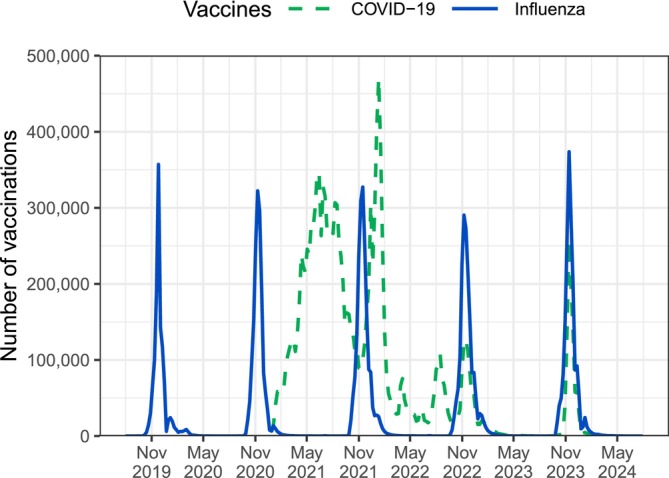
COVID‐19 and influenza vaccinations in Finland by calendar week, August 2019 to May 2024.

To contain the spread of COVID‐19, Finland adopted several nonpharmaceutical interventions and a comprehensive COVID‐19‐specific vaccination strategy. The three nonpharmaceutical interventions that likely also had a substantial impact on the transmission of other respiratory diseases, such as influenza, were social distancing (enforced from March 2020 to June 2021, with interruptions) [15, 16], the use of face masks (recommended from August 2020 to April 2022) [17, 18], and the restriction of international travel (enforced from March 2020 to June 2022) [15, 19]. The vaccination campaign started in late December 2020 (Figure 2) with prioritization of health care workers, the elderly, and people at high risk of severe COVID‐19 [20]. By August 2021, a primary series of mostly two doses had been offered free of charge to all people aged 12 years and above [21]. After that, up to three booster doses [22] and seasonal boosters in 2022–2023 [23] and 2023–2024 [24] were recommended for selected risk groups.

### 3.2 Clinical Influenza Surveillance

Figure 1B,C shows the weekly number of laboratory‐confirmed, medically attended, and hospitalized influenza cases from August 2019 to May 2024. The 2019–2020 influenza epidemic started in November 2019 and ended abruptly in March 2020. A total of 10,237 influenza A and 2271 influenza B cases were laboratory confirmed between August 2019 and July 2020. In addition, there were 5914 primary health care visits due to ILI during this last prepandemic season (Figure 1C).

Throughout 2020–2021, the influenza activity was unusually low. Only a few sporadic cases (34 influenza A and 22 influenza B infections) were detected (Figure 1C).

After a small wave of influenza infections at the end of 2021, the 2021–2022 epidemic peaked exceptionally late, that is, in the first week of May, and was very short, lasting only 7 weeks. A total of 6976 influenza A cases were laboratory confirmed; 476 of which were hospitalized with respiratory symptoms. There was almost no influenza B activity, as only 98 infections were detected (Figure 1B,C).

The subsequent two postpandemic influenza epidemics were similar in shape, duration, and intensity (Figure 1C). Both in 2022–2023 and 2023–2024, a big wave of influenza A infections (13,728 and 10,257 cases, respectively) peaked in the last weeks of December and was later followed by a small wave of influenza B infections (2172 and 1210 cases, respectively). The number of primary health care visits due to ILI increased to 16,698 and 16,684, respectively. In 2022–2023, 1485 influenza A and 179 influenza B cases were hospitalized. Similarly, in 2023–2024, there were 1406 influenza A and 109 influenza B cases that required hospitalization (Figure 1B).

Documented influenza and SARS‐CoV‐2 coinfections have been rare. Since 2021–2022, a few hundred coinfections were detected each season (259 in 2021–2022, 382 in 2022–2023, and 346 in 2023–2024), but their proportion among all influenza infections has remained less than 5%.

**FIGURE 3:**
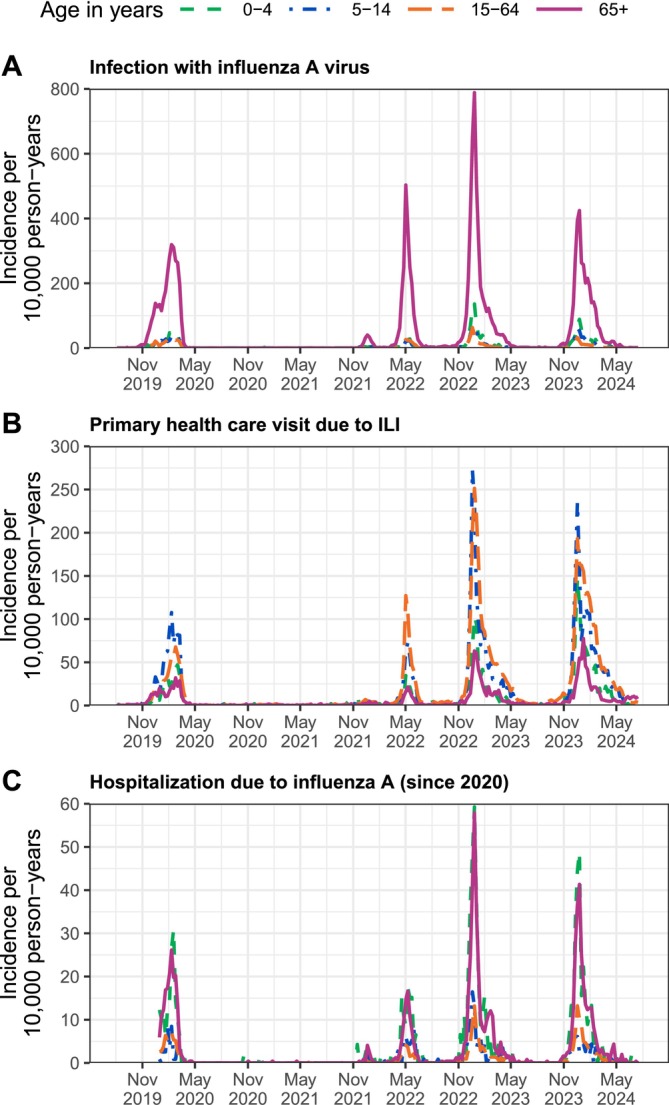
Influenza incidence per 10,000 person‐years in Finland by age group and calendar week, August 2019 to May 2024. (A) Infection with influenza A virus; (B) Primary health care visit due to ILI; (C) Hospitalization due to influenza A (since 2020). Adapted from [5, 6].

Figure 3 shows the weekly influenza incidence rate in 0‐to‐4‐year‐olds, 5‐to‐14‐year‐olds, 15‐to‐64‐year‐olds, and those aged 65 years and above. Excluding 2020–2021, the mean incidence of laboratory‐confirmed influenza A infection ranged between 44.1 and 83.7 per 10,000 person‐years in the elderly population but remained below 13 per 10,000 person‐years in the other three age groups (Figure 3A). School‐age children and working‐age adults bore the greatest burden of medically attended mild disease. In 2022–2023, for example, the mean incidence of primary health care visits due to ILI was 31.0 and 38.0 per 100,000 person‐years in 5‐to‐14‐year‐olds and 15‐to‐64‐year‐olds, respectively, whereas it was smaller than 13 per 10,000 person‐years in the other two age groups (Figure 3B). By contrast, 0‐to‐4‐year‐old children and the elderly population bore the greatest burden of severe disease. In 2022–2023, the mean incidence of hospitalization due to influenza A in these two age groups was 5.8 per 100,000 person‐years (Figure 3C). However, on average, young children were hospitalized for only one night (interquartile range: 1; 2), whereas people aged 65 years and above were hospitalized for four nights (interquartile range: 2; 7).

### 3.3 Virological Influenza Surveillance

Through all seasons, the number of respiratory specimens collected by outpatient sentinel sites was below the expected three to five specimens per week: 157 specimens were received in 2019–2020, 166 in 2021–2022, 177 in 2022–2023, and 217 in 2023–2024. Similarly, the number of specimens sent by intensive care units was below the requested quota: 74 specimens were received in 2019–2020, 0 in 2021–2022, 22 in 2022–2023, and 17 in 2023–2024.

Table 1 shows the distribution of influenza A virus subtypes and influenza B virus lineages in influenza‐positive specimens. In 2019–2020, influenza A(H1)pdm09, influenza A(H3), and influenza B/Victoria viruses circulated in the population. The distribution of influenza A virus subtypes varied greatly between the types of sites where the specimens were obtained. Whereas 70% (53/76) and 14% (11/76) of the influenza‐positive specimens collected at the sentinel sites and intensive care units were positive for influenza A(H1)pdm09 and influenza A(H3), respectively, the corresponding figures for specimens sent by clinical microbiology laboratories were 31% (14/45) and 47% (21/45). Influenza A(H3) dominated in 2021–2022 and 2022–2023 and was found in at least 65% (157/243) of those seasons' influenza‐positive specimens, whereas influenza A(H1)pdm09 was the most common subtype in 2023–2024.

**TABLE 1:** Distribution of influenza A virus subtypes and influenza B virus lineages in Finland.

	2019–2020	2020–2021	2021–2022	2022–2023	2023–2024
Sentinel sites and intensive care units					
A(H1)pdm09	53 (70%)	—	0 (0%)	7 (11%)	26 (51%)
A(H3)	11 (14%)	—	58 (100%)	54 (82%)	14 (27%)
B/Victoria	12 (16%)	—	0 (0%)	5 (8%)	11 (22%)
B/Yamagata	0 (0%)	—	0 (0%)	0 (0%)	0 (0%)
Clinical microbiology laboratories					
A(H1)pdm09	14 (31%)	—	2 (1%)	36 (15%)	1096 (76%)
A(H3)	21 (47%)	—	195 (97%)	157 (65%)	183 (13%)
B/Victoria	9 (20%)	—	3 (1%)	50 (21%)	160 (11%)
B/Yamagata	1 (2%)	—	1 (0%)	0 (0%)	2 (0%)

Sporadically, also influenza B/Victoria viruses were detected (Table 1). However, none of the 717 sentinel specimens nor any of the 113 specimens from intensive care units tested positive for the B/Yamagata lineage. The three B/Yamagata‐positive specimens sent by clinical microbiology laboratories originated from children who had recently been vaccinated with the live‐attenuated influenza vaccine containing that specific lineage.

### 3.4 Influenza Vaccine Uptake and Vaccination Coverage

From 2019–2020 to 2021–2022, the total number of influenza vaccinations given each season had steadily increased from 1,229,294 to 1,902,073. In the subsequent two postpandemic seasons, 1,752,730 and 1,777,842 influenza vaccinations took place. The most frequently used vaccine brand was Vaxigrip Tetra (Sanofi), an inactivated vaccine, even though its share among all brands has gradually decreased from 92% (1,135,264/1,229,294) in 2019–2020 to 85% (1,514,375/1,777,842) in 2023–2024. Other frequently used brands were Fluenz Tetra (AstraZeneca), a live‐attenuated vaccine, with a share of 4% (73,650/1,777,842) in 2023–2024 and, since 2020–2021, Fluarix Tetra (GlaxoSmithKline), another inactivated vaccine, which was exclusively used by occupational and private providers, with a share of 9% (163,805/1,777,842) in 2023–2024.

Figure 2 shows the weekly number of seasonal influenza vaccinations from August 2019 to May 2024 as well as the weekly number of COVID‐19 vaccinations since December 2020. During the pandemic, coadministration of influenza and COVID‐19 vaccines was rare. In 2022–2023 and 2023–2024, however, the two vaccination campaigns were timely aligned and 36% (628,990/1,752,730) and 47% (832,088/1,777,842) of the influenza vaccinations were given within 14 days before or after a COVID‐19 vaccination and, of these, about 95% (590,161/628,990 and 808,526/832,088) on the same day.

In all of the past five seasons, most influenza vaccinations took place in November (Figure 2). From 2020–2021 to 2022–2023, the vaccination campaign started in the second half of October. The other two seasons' campaigns started later, at the beginning of November. The vaccination campaigns in 2020–2021 and 2023–2024 were relatively short, with more than 95% (1,568,009/1,609,810 and 1,701,187/1,777,842) of the vaccines administered by the end of December. The other campaigns continued until January.

**FIGURE 4:**
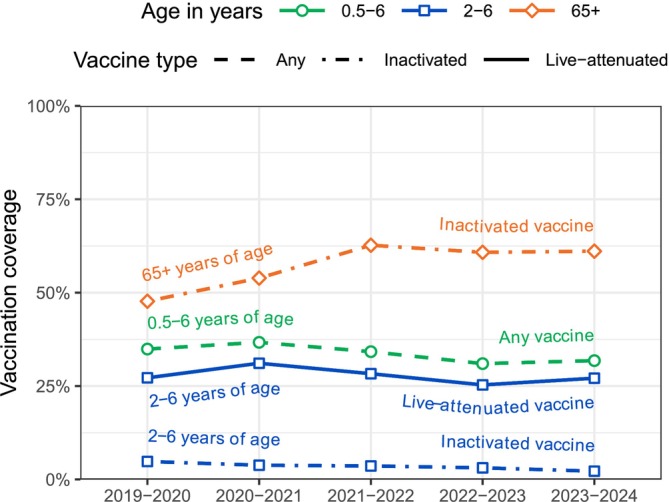
Influenza vaccination coverage in Finland by age group and season. Adapted from [25].

Figure 4 shows the influenza vaccination coverage for the two age groups universally included in the national vaccination program, that is, children younger than 7 years and adults aged 65 years and above, by season. Although the percentage of vaccinated young children, ranging between 37% (126,984/346,344) in 2020–2021 and 31% (100,387/323,614) in 2022–2023, was roughly constant over the past five seasons, the percentage of vaccinated elderly people increased substantially from 48% (577,404/1,211,732) in 2019–2020 to 63% (787,771/1,255,644) in 2021–2022 and has remained above 60% (773,450/1,272,815 and 796,393/1,303,097) since then. In 2‐to‐6‐year‐old children, the vaccination coverage of the inactivated, injectable vaccine has continuously decreased from 5% (13,628/285,039) in 2019–2020 to 2% (5420/245,387) in 2023–2024, indicating a strong preference for the live‐attenuated nasal spray vaccine, although both vaccines have been equally available through the national vaccination program.

### 3.5 Influenza Vaccine Effectiveness

Table 2 shows the estimates of influenza vaccine effectiveness against laboratory‐confirmed infection with any influenza virus and hospitalization due to any influenza virus in the study cohorts of approximately 300,000 children younger than 7 years and one million adults aged 65 years and above. In both age groups, the effectiveness against hospitalization was similar to that against infection and differed by a maximum of five percentage points. The estimates are mainly determined by the vaccine effectiveness against influenza A. In 2019–2020 and 2022–2023, when separate effectiveness analyses against the two virus types were deemed reliable, the vaccine effectiveness against influenza B was higher than that against influenza A.

**TABLE 2:** Influenza vaccine effectiveness in Finland by age group and season.

		2019–2020	2020–2021	2021–2022	2022–2023	2023–2024
Children aged 0.5–6 years	Cohort size	317,060	—	302,850	304,310	296,434
Infection with influenza virus	Number of cases (vaccinated; unvaccinated)	1504 (1283; 221)	—	437 (349; 88)	1191 (1102; 89)	1081 (931; 150)
	Vaccine effectiveness (95% confidence interval)	61% (55%; 66%)	—	44% (30%; 56%)	69% (61%; 75%)	45% (34%; 54%)
Hospitalization due to influenza	Number of cases (vaccinated; unvaccinated)	—	—	—	139 (129; 10)	104 (92; 12)
	Vaccine effectiveness (95% confidence interval)	—	—	—	68% (38%; 83%)	50% (7%; 73%)
Adults aged 65 years and above	Cohort size	1,153,474	—	1,277,958	1,299,965	1,314,721
Infection with influenza virus	Number of cases (unvaccinated; vaccinated)	1824 (1160; 664)	—	997 (337; 660)	3784 (2014; 1770)	3497 (1869; 1628)
	Vaccine effectiveness (95% confidence interval)	24% (15%; 33%)	—	‐5% (−23%; 10%)	37% (32%; 41%)	41% (36%; 46%)
Hospitalization due to influenza	Number of cases (unvaccinated; vaccinated)	—	—	—	1140 (625; 515)	1118 (601; 517)
	Vaccine effectiveness (95% confidence interval)	—	—	—	42% (34%; 50%)	40% (30%; 48%)

The lowest vaccine effectiveness was observed in 2021–2022, when by the end of the epidemic, which peaked exceptionally late, vaccination had prevented 44% (95% confidence interval: 30%; 56%) of the otherwise expected laboratory‐confirmed infections in vaccinated children but had seemingly prevented no such infections in the vaccinated elderly population (Table 2). However, also in the elderly population, vaccination demonstrated a protective effect—during the first 14–90 days after vaccination, when the vaccine effectiveness was estimated at 40% (95% confidence interval: 7%; 61%). This was also roughly the end‐of‐season vaccine effectiveness level observed in the subsequent two postpandemic seasons in that age group, though the narrow confidence intervals, covering only about 10 percentage points, indicate more precise estimates (Table 2).

The highest vaccine effectiveness was observed in children younger than 7 years in 2022–2023, when by the end of the epidemic vaccination had reduced the incidence of laboratory‐confirmed infection in vaccinated children by 69% (95% confidence interval: 61%; 75%) (Table 2). In the subgroup of 2‐to‐6‐year‐olds, the corresponding effectiveness of the inactivated vaccine was estimated at only 32% (95% confidence interval: −26%; 64%) but that of the live‐attenuated vaccine, which most of the vaccinated children had received, was estimated at solid 69% (95% confidence interval: 61%; 76%). In 2019–2020, the other season when separate effectiveness analyses of the two vaccines were deemed reliable, the difference was smaller; the effectiveness of the inactivated and the live‐attenuated vaccines was estimated at 52% (95% confidence interval: 37%; 64%) and 66% (95% confidence interval: 58%; 72%), respectively.

## 4 Discussion

The COVID‐19 pandemic had a significant impact on the epidemiology of influenza in Finland. The 2019–2020 influenza epidemic ended abruptly in March 2020 with the introduction of the nonpharmaceutical COVID‐19 containment measures. During the 2020–2021 season, influenza was largely absent. The 2021–2022 influenza epidemic was very short and peaked exceptionally late, in May 2022, immediately after the recommendation to use face masks was lifted. In addition, no evidence of circulation of influenza B/Yamagata viruses was found between 2019 and 2024, indicating the elimination of this lineage. The threat of COVID‐19 as a fast‐spreading severe respiratory disease and the success of the COVID‐19 vaccination campaign likely raised the awareness of influenza and influenza vaccination especially among elderly people, whose influenza vaccination coverage increased during the COVID‐19 pandemic. Furthermore, coadministration of influenza and COVID‐19 vaccines had no noticeable, clinically relevant impact on the influenza vaccine effectiveness. However, it appears that the COVID‐19 pandemic indirectly affected the end‐of‐season effectiveness of influenza vaccination in the elderly population negatively by delaying the 2021–2022 influenza epidemic until the vaccine‐induced protection had waned.

Overall, the epidemiology of influenza in Finland was comparable to that in the rest of Europe during the COVID‐19 pandemic [26, 27, 28]. The influenza activity decreased significantly in March 2020 in most countries, and only 10 out of 21,442 sentinel specimens from across the continent tested positive for influenza in 2020–2021 [26, 27]. However, the delayed 2021–2022 epidemic was particularly late in Finland, where the epidemic peaked about a month after it had peaked in other European countries [28]. This likely affected the vaccine effectiveness, especially in elderly people whose vaccine‐induced immunity wanes towards the end of a season due to immunosenescence. When studying only the first 90 postvaccination days, the Finnish estimates are in line with other interim reports, but the end‐of‐season estimates are considerably lower [28, 29]. Furthermore, no evidence of circulation of influenza B/Yamagata viruses has been found across Europe since 2020–2021 [27, 28, 30, 31]. In 2022–2023, the first postpandemic season, the influenza activity, and vaccine effectiveness were similar in Finland and the rest of the continent [30]. In 2023–2024, European sentinel surveillance data indicated a relatively short season, whereas the Finnish clinical surveillance data showed no such anomaly [31]. In comparison to the few other countries reporting influenza vaccination coverage in children, Finland performs moderately well [32]. However, the vaccination coverage in the elderly population still does not reach the 75% target set by the Council of the European Union [33], although it increased to 63% during the pandemic and is thus in line with the European median of 59% for 2020–2021 [32].

The COVID‐19 pandemic dealt a major blow to traditional influenza surveillance in Finland. During the pandemic, individuals with respiratory symptoms were directed to centralized coronavirus sampling centers, which led to the collapse of respiratory virus surveillance. After the pandemic, it has been challenging to recruit sentinel sites to voluntarily participate in respiratory virus surveillance largely due to reduced resources in health care and the demanding nature of the surveillance process, particularly in recruiting participants, which includes obtaining written consent. Clinical microbiology laboratories were therefore requested to send positive respiratory specimens for characterization. However, the interpretation of the sum of these results has remained difficult: The virus distribution differs between the types of sites where the samples were collected, the numbers from sentinel sites are neither representative nor large enough to infer any trends within a season, and the background of the specimens received from clinical laboratories is unknown. Thus, THL has proposed an amendment of the national Communicable Diseases Act obligating each of the 21 wellbeing services counties in Finland to nominate one sentinel site within their borders to actively participate in the sentinel surveillance of influenza and other respiratory viruses to ensure representativeness.

One positive side effect of the COVID‐19 pandemic was an improvement in the quality of the available register data. In particular, the acquisition of hospitalization data in near real‐time and the acquisition of data on private primary health care visits were tackled early on and achieved within a year. As a result, however, some of the observed temporal trends, such as the increase in primary health care visits due to ILI (Figure 1C), cannot be exclusively attributed to a change in disease burden (e.g., due to attenuation of immunity following the absence of influenza in 2020–2021) or health‐seeking behavior (e.g., due to COVID‐19‐related increased awareness of respiratory infections in general) [34]. Nevertheless, because influenza vaccinations for both young children and elderly people are covered by the national vaccination program, which is carried out in the public sector, it is very likely that the vaccination coverage has indeed improved among the latter. Future analyses of the impact of the influenza vaccination program in Finland should also focus on the rest of the population, especially pregnant women, social and health care workers, and people at risk of severe influenza disease because of an underlying chronic illness or immunosuppressive treatment supporting the implementation of the European Union's health technology assessment regulation on national and European level [35].

The biggest challenge in interpreting the presented data arises from the fact that the age‐specific likelihood of laboratory testing when suspecting an infection is unknown. The total number of laboratory‐confirmed influenza infections and the total number of primary health care visits due to ILI appear to match well (Figure 1C), but the age‐specific influenza incidence figures show an inconsistent distribution of infections and ILI across age groups (Figure 3). ILI frequently diagnosed in primary care in school‐age children and working‐age adults seems to be rarely laboratory confirmed, probably because patients have only mild symptoms that started more than 48 h ago. Interestingly, the high incidence of infections in the elderly population is reflected neither in the frequency of primary health care visits nor in the frequency of hospitalization. The best explanations for this finding might be that elderly people with an influenza infection are tested at a lower threshold and with broader spectrum of symptoms than the rest of the population, for example, in nursing homes, and that, because they might present only with unspecific symptoms, such as fever, delirium, or exacerbation of chronic comorbidities [4], they do not fall under the case definitions that focus on respiratory illness. The latter might also explain why the presented estimates of vaccine effectiveness against infection and hospitalization are so similar, although the influenza vaccine is known to lower the severity of disease but not necessarily prevent infection [36]; both case definitions may capture similarly severe symptoms, with hospitalization focusing solely on respiratory symptoms.

Because the age‐specific likelihood of laboratory testing when suspecting an infection and, thus, the sensitivity of the register‐based outcome measurement of influenza infection are unknown and likely imperfect, the estimation of vaccine effectiveness against infection cannot be adjusted for outcome measurement errors, and the reader is directed to a prior publication for a deeper exploration of bias due to nonsensitive observation of outcomes in time‐to‐event data [37]. By contrast, standard diagnostic practices in hospitals, such as laboratory testing and the use of ICD‐10 codes, ensure a comprehensive representation of the influenza disease burden due to severe respiratory illness through register data, largely consistent with common syndromic case definitions [1, 8]. Nevertheless, it should be stressed that the methods underlying the presented effectiveness estimates do not address the issues of potential mismatches between circulating and vaccine strains, confounding in children, and waning vaccine effectiveness in elderly people comprehensively. These require separate, elaborate studies beyond the scope of routine surveillance. The recently established European Vaccine Monitoring Platform facilitates and coordinates such studies and has, among other things, the assessment of brand‐specific influenza vaccine effectiveness on its agenda [38].

## 5 Conclusion

The COVID‐19 pandemic revealed the strength of a hybrid influenza surveillance system, such as the Finnish one. Although resource‐intensive virological surveillance temporarily collapsed, the largely automated register‐based clinical surveillance continued. In addition, the COVID‐19 pandemic had a significant impact on the epidemiology of influenza in Finland, but only two effects have so far persisted also in the postpandemic period: first, the elimination of the influenza B/Yamagata lineage supporting the World Health Organization's recommendation to simplify the influenza vaccine composition by not including that lineage anymore [39] and, second, the improved influenza vaccination coverage in the elderly population. The 75% target vaccination coverage set by the Council of the European Union [33] remains a goal to achieve in future seasons. Another ambitious goal is to improve participation in sentinel surveillance and thus to strengthen the Finnish influenza surveillance system.

## Author Contributions

**Ulrike Baum:** conceptualization, formal analysis, writing – original draft. **Niina Ikonen:** investigation, writing – review and editing. **Oskari Luomala:** investigation, writing – review and editing. **Eero Poukka:** supervision, writing – review and editing. **Tuija Leino:** supervision, writing – review and editing. **Hanna Nohynek:** conceptualization, supervision, writing – review and editing.

## Conflicts of Interest

Eero Poukka participated in the IMI‐funded PROMISE project until April 2024, has received a personal grant from the Finnish Medical Foundation, and has recently been involved in an estate that holds shares of Astra Zeneca. Hanna Nohynek was colead of the IMI‐funded DRIVE consortium until June 2023. Hanna Nohynek is the principal investigator of a THL‐sponsored immunogenicity study on repeated influenza vaccinations among health care workers, a NITAG member, and the chairman of WHO SAGE. All other authors, that is, Ulrike Baum, Niina Ikonen, Oskari Luomala, and Tuija Leino, have no conflicts of interest to disclose.

## Peer Review

The peer review history for this article is available at https://www.webofscience.com/api/gateway/wos/peer‐review/10.1111/irv.70131.

## Data Availability

In accordance with Finnish law, the authors are not permitted to share individual‐level register data. However, this surveillance report also incorporates data published online by the Finnish Institute for Health and Welfare under a Creative Commons Attribution 4.0 International License. Links to these publicly available data sources are provided in the manuscript.
